# Supplementary material for: Global Gene Expression of Post-Senescent Telomerase-Negative ter1Δ Strain of Ustilago maydis
Source: J Fungi (Basel). 2023 Aug 31;9(9):896. doi: 10.3390/jof9090896 (PMC10532341; doi:10.3390/jof9090896)
Supplement: Supplementary file 1 [file jof-09-00896-s001.zip › Supplementary final/Figure S1.pdf]

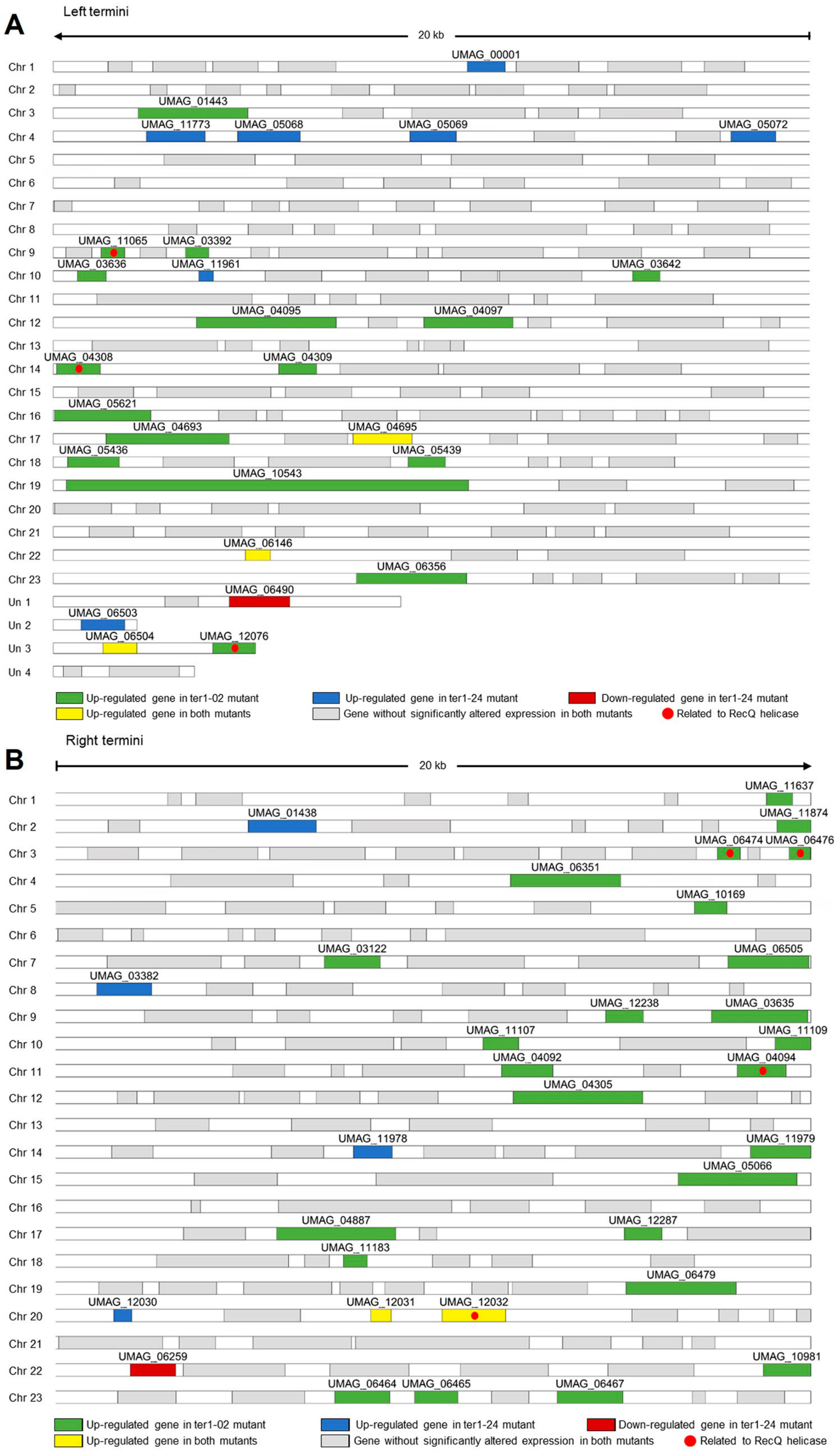

Figure S1. Transcriptional landscape of chromosomal ends of *U. maydis*.
(A) Distribution of DEGs located in the 20 kb near the left end of the genome.
(B) Distribution of DEGs located in the 20 kb near the right end of the genome.
